# Enhancing Decision Support for Vector-Borne Disease Control Programs—The Disease Data Management System

**DOI:** 10.1371/journal.pntd.0004342

**Published:** 2016-02-18

**Authors:** Edward K. Thomsen, Rinki M. Deb, Sophie Dunkley, Marlize Coleman, Geraldine Foster, Miguel Orlans, Michael Coleman

**Affiliations:** Department of Vector Biology, Liverpool School of Tropical Medicine, Liverpool, United Kingdom; Centers for Disease Control and Prevention, UNITED STATES

## Introduction

Data is at the core of any successful vector-borne disease control or elimination activity. At the early stages of control, monitoring data can help prioritize limited funding and resources to maximize impact. During the pre-elimination and elimination phases, surveillance data itself becomes the primary intervention by quickly identifying persistent transmission [1]. In addition, it has been identified that spatial decision-support tools will be crucial to integrate with health information systems (HIS) as countries strive for elimination [2].

The Disease Data Management System (DDMS) is a tool designed to meet the data management and decision-support needs of vector-borne disease control programs as they transition through control to elimination. The development and functionality of the DDMS has been described elsewhere [3], and particular advantages and disadvantages are highlighted in Box 1. Here, we describe the implementation and impact of the system in disease-endemic countries, user feedback, and future challenges.

**Box 1. Advantages and Disadvantages of the DDMS**

**Advantages**
- High configurability means that the system can be adjusted for any vector-borne disease control program.
- Capable of supporting decision making from control through elimination phases.
- Decision support, reporting, and spatial visualization components for multiple diseases integrated into a single tool.
- Query builders mean that the user is not limited to pre-defined reports but can easily create custom queries on demand.

**Disadvantages**
- High configurability means that well-trained administrators are required.
- System versatility sometimes causes untrained users to experience the DDMS as being overly complex.
- Direct mobile data capture not currently integrated.

## System Implementation

**Fig 1:**
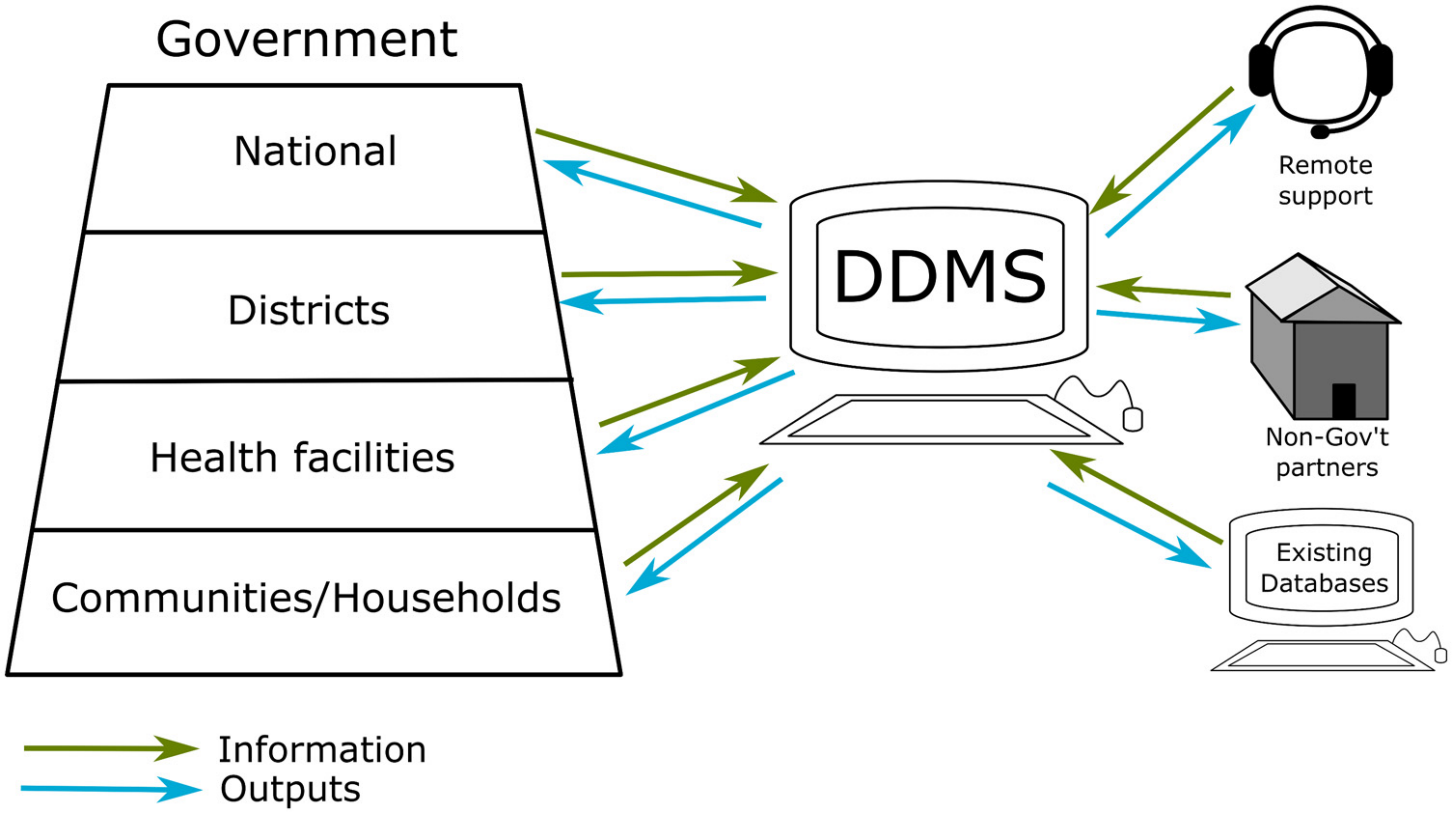
A typical setup for the implementation of the DDMS. The server itself can sit at any level with a reliable internet connection, but usually it is at the national level (Ministry of Health) or with a nongovernment partner. It can be offsite on an international server and is therefore compatible with cloud computing.

The DDMS has been implemented in seven countries in Africa and Asia (Table 1). All countries have employed a similar system architecture in which the database is accessible via the internet and there is bidirectional flow of data and outputs at all organizational levels (Fig 1).

**Table 1:** Description of where the DDMS is currently being implemented, for what purpose, and by what organization.

Country	Disease	DDMS Modules Used	Implementing Organization
Benin	Malaria	case surveillance (passive)	Ministry of Health
Ghana	Malaria	entomological surveillance	The President’s Malaria Initiative (PMI)-funded Africa Indoor Residual Spray Project (AIRS)
Ethiopia	Malaria	entomological surveillance	The PMI-funded AIRS project
Zambia	Malaria	entomological surveillance, Indoor Residual Spray (IRS) planning and monitoring	The PMI-funded AIRS project and Ministry of Health
Mali	Malaria	entomological surveillance	The PMI-funded AIRS project
Equatorial Guinea	Malaria	entomological surveillance, case surveillance (active and passive), IRS planning and monitoring, indicator surveys, long-lasting insecticidal net (LLIN) distribution, larval source management, form builder for custom forms	Bioko Island Malaria Control Project
India	Visceral Leishmaniasis	entomological Surveillance, IRS monitoring	Rajendra Memorial Research Institute

Overall, the primary impact in most countries has been increased accessibility of data and therefore more informed decision making. This is most apparent in Zambia, where the Insecticide Resistance Management Technical Working Group uses DDMS outputs, in the form of maps and reports, to inform decisions related to management of insecticide resistance [4]. The implementation in Zambia, although a good example of how the DDMS can impact decision making, has not been without its challenges. Since November 2014, the DDMS has not been used, primarily because of a loss of momentum as the responsibility of maintaining the system shifted between malaria control partners. This has highlighted the need to bolster the capacity of related systems (organizational data culture, data collection procedures, IT infrastructure) to support DDMS implementations.

The prospect of increased data accessibility prompted the Africa IRS (AIRS) project, funded by the President’s Malaria Initiative, to adopt the DDMS after initial trials in Zambia and Ethiopia [5]. This will involve the deployment of the DDMS as the entomological database in eight AIRS countries in sub-Saharan Africa. The DDMS in each country can be accessed by stakeholders nationally and internationally, which has made comparison of data at the international level more feasible.

## User Feedback

DDMS users were asked to complete an anonymous online survey to assess perceptions on usefulness, usability, and comparison with previous data-handling methods (S1 and S2 Appendices). Responses were received from ten individuals in five countries. The results from this survey have highlighted the strengths of the system as well as aspects that are underutilized (Table 2). For example, nearly everyone felt that the DDMS was both useful and easy to use. However, some components, such as the mapping module and reporting tool, were consistently highlighted as difficult to use. In addition, job functions related to these components were highlighted as areas where the DDMS was not very useful. Some of these issues can be addressed with more rigorous training, while others can be addressed with a redesign of the user interface. Both of these aspects will be important to focus on to ensure that the system is utilized to its fullest capacity.

**Table 2:** User feedback.

Question or Statement	Percent agreement (responses)	Additional feedback and information
I find the DDMS useful in my job	80% (10)	+ data entry, checking data accuracy, modifying and/or cleaning data, summarizing and/or tabulating data, generating reports - creating charts and/or graphs, making maps, making programmatic decisions
I find the DDMS easy to use	78% (9)	+ user interface, data entry screens, query builders, data import functionality - mapping module, reporting tool, information trees
Would you be interested in using the DDMS to handle other data?	90% (10)	
Compared to previous methods of handling raw data, the DDMS is better in terms of:		
Data quality	80% (10)	
Access to the data	67% (9)	
Manipulation of the data	44% (9)	
Ability to easily summarize the data	78% (9)	
Speed with which data-related tasks are completed	40% (10)	

Eighty percent of users felt that the DDMS improved data quality compared to previous methods (usually Excel or Access databases). Processes that occur outside of the DDMS, such as data collection and entry procedures, influence some characteristics of quality data, such as timeliness and completeness. However, accuracy, accessibility, and consistency are data characteristics that are likely improved by using the DDMS and are primarily facilitated by ontological trees specifying which data can be entered as well as query builders that improve data accessibility. A thorough evaluation of data quality is necessary to confirm users’ perceptions.

## Challenges Remaining

Positive user feedback, combined with the documented impact of this tool on decision making, reinforces the need to support the adoption of decision-support tools in disease control programs. However, experience with the DDMS has highlighted a primary challenge in achieving this goal: information systems do not exist in silos. They need to be underpinned by an organizational culture that values data use, data collection, and transfer processes that maximize reliability, as well as infrastructure to support their deployment.

If this challenge is addressed, the DDMS possesses several unique features that could support global goals of elimination and control. First, as a multidisease system, the DDMS can facilitate integration of vector control programs, which can bolster neglected tropical disease elimination efforts [6]. Second, because of standardized data formats, the DDMS can facilitate cross-border collaboration and collective decision making. The feasibility of this has already been demonstrated through the AIRS project. Third, the DDMS has a sophisticated alert system that responds in real time as individual cases are entered. As control programs progress from the control to elimination phases, this will be a crucial feature that will allow rapid and focal response to outbreaks [7]. Lastly, the DDMS uses open-source technology that can integrate with other widely-used HIS, such as District Health Information Software 2 (DHIS2). The DDMS can augment current DHIS2 functionality by providing unique tools that support daily decision-making in vector-borne disease control programs. Data could therefore enter the system via the DDMS and be shuttled into a database like the DHIS2 for integration with higher-level health system data.

Moving forward, it will be necessary to rigorously assess the DDMS as any other component of an HIS to determine the technical, behavioral, and organizational determinants of its effect [8]. While user feedback is useful in determining strengths and weaknesses, future work needs to move beyond perceptions to see if the DDMS adds value to the overall HIS. In order to assure its sustainability, the DDMS user community must be able to connect with one another to seek guidance. This will decrease the reliance on a central organization to provide support. In addition, it will be necessary to secure development funding to ensure that the software continues to stay up to date with current technology.

## Supporting Information

S1 Appendix DDMS User Survey—English. (PDF)

S2 Appendix DDMS User Survey—French. (PDF)
