# Supplementary material for: Enhancing Decision Support for Vector-Borne Disease Control Programs—The Disease Data Management System
Source: PLoS Negl Trop Dis. 2016 Feb 18;10(2):e0004342. doi: 10.1371/journal.pntd.0004342 (PMC4758655; doi:10.1371/journal.pntd.0004342)
Supplement: S1 Appendix — (PDF) [file pntd.0004342.s001.pdf]

# DDMS survey

1. **Which of the following best describes your job function?**
   *Mark only one oval.*

   - Entomologist
   - Information Technology
   - Management
   - Data entry
   - Data management
   - M&E
   - Other: ____________

2. **What country do you work in?**
   *Mark only one oval.*

   - Benin
   - Ethiopia
   - Equatorial Guinea
   - Ghana
   - India
   - Mali
   - Zambia
   - Other: ____________

3. **How often do you use the DDMS?**
   *Mark only one oval.*

   - Never
   - Rarely
   - Regularly

4. **Using the DDMS in my job enables me to accomplish tasks more quickly**
   1: Strongly disagree; 2: Somewhat disagree; 3: Neither agree nor disagree; 4: Somewhat agree; 5: Strongly agree
   *Mark only one oval.*

| | 1 | 2 | 3 | 4 | 5 | |
|---|---|---|---|---|---|---|
| Strongly disagree | | | | | | Strongly agree |

5. **Which tasks does the DDMS enable you to accomplish more quickly?**
Click "Not Applicable" if you do not need to complete these tasks in your current position
*Mark only one oval per row.*

| | Yes | No | Not Applicable |
|---|---|---|---|
| Entering data | | | |
| Checking data accuracy | | | |
| Modifying/cleaning data | | | |
| Querying/Summarizing/Tabulating data | | | |
| Creating charts and graphs | | | |
| Generating reports | | | |
| Making maps | | | |
| Making programmatic decisions | | | |
| Other | | | |

6. **If yes to "Other", describe**

7. **I find the DDMS useful in my job**
1: Strongly disagree; 2: Somewhat disagree; 3: Neither agree nor disagree; 4: Somewhat agree; 5: Strongly agree
*Mark only one oval.*

| | 1 | 2 | 3 | 4 | 5 | |
|---|---|---|---|---|---|---|
| Strongly disagree | | | | | | Strongly agree |

8. **Which tasks do you find the DDMS is useful in completing?**
Click "Not Applicable" if you do not need to complete these tasks in your current position
*Mark only one oval per row.*

| | Yes | No | Not Applicable |
|---|---|---|---|
| Entering data | | | |
| Checking data accuracy | | | |
| Modifying/cleaning data | | | |
| Querying/Summarizing/Tabulating data | | | |
| Creating charts and graphs | | | |
| Generating reports | | | |
| Making maps | | | |
| Making programmatic decisions | | | |
| Other | | | |

9. **If yes to "Other", describe**

10. **I find the DDMS easy to use**

1: Strongly disagree; 2: Somewhat disagree; 3: Neither agree nor disagree; 4: Somewhat agree; 5: Strongly agree

*Mark only one oval.*

| | 1 | 2 | 3 | 4 | 5 | |
|---|---|---|---|---|---|---|
| Strongly disagree | ◯ | ◯ | ◯ | ◯ | ◯ | Strongly agree |

11. **Which components of the DDMS are easy to use?**

Click "Not Applicable" if you do have not used a particular component

*Mark only one oval per row.*

| | Yes | No | Not Applicable |
|---|---|---|---|
| General user interface | ◯ | ◯ | ◯ |
| Data entry screens | ◯ | ◯ | ◯ |
| Query builders | ◯ | ◯ | ◯ |
| Mapping module | ◯ | ◯ | ◯ |
| Reporting tools | ◯ | ◯ | ◯ |
| Data import functionality | ◯ | ◯ | ◯ |
| Geography tree | ◯ | ◯ | ◯ |
| Term tree | ◯ | ◯ | ◯ |
| Security functionality | ◯ | ◯ | ◯ |
| Form builder | ◯ | ◯ | ◯ |
| Localisation (changing languages) | ◯ | ◯ | ◯ |

12. **What components of the DDMS do you find most useful?**

Please place a check mark next to the TOP THREE components

*Check all that apply.*

- [ ] Data entry screens
- [ ] Query builders
- [ ] Mapping module
- [ ] Reporting tools
- [ ] Data import functionality
- [ ] Geography tree
- [ ] Term tree
- [ ] Security functionality
- [ ] Form builder
- [ ] Localisation (changing languages)
- [ ] Other: ..........................................

13. **What components of the DDMS do you think could be improved to make them more useful?**
Please place a check mark next to the TOP THREE components
*Check all that apply.*

- [ ] Data entry screens
- [ ] Query builders
- [ ] Mapping module
- [ ] Reporting tools
- [ ] Data import functionality
- [ ] Geography tree
- [ ] Term tree
- [ ] Security functionality
- [ ] Form builder
- [ ] Localisation (changing languages)
- [ ] Other: ..........................................

14. **Please describe how the components you checked above could be improved.**

..........................................

..........................................

..........................................

..........................................

..........................................

15. **How were you organizing your raw data prior to using the DDMS?**
*Check all that apply.*

- [ ] Excel spreadsheets
- [ ] Access database
- [ ] Other database
- [ ] Paper forms only
- [ ] Other: ..........................................

16. **How does the DDMS compare to this other method in terms of...?**
*Mark only one oval per row.*

| | Better | The same | Worse | I don't know |
|---|---|---|---|---|
| Data quality | ◯ | ◯ | ◯ | ◯ |
| Access to the data | ◯ | ◯ | ◯ | ◯ |
| Manipulation of the data | ◯ | ◯ | ◯ | ◯ |
| Ability to easily summarize the data | ◯ | ◯ | ◯ | ◯ |
| Speed with which data-related tasks are completed | ◯ | ◯ | ◯ | ◯ |

17. **Would you be interested in using the DDMS to handle other data?**
*Mark only one oval.*

- Yes
- No

18. **Why or why not?**

19. **Any additional comments?**

Powered by
Google Forms
