# Supplementary material for: Enhancing Decision Support for Vector-Borne Disease Control Programs—The Disease Data Management System
Source: PLoS Negl Trop Dis. 2016 Feb 18;10(2):e0004342. doi: 10.1371/journal.pntd.0004342 (PMC4758655; doi:10.1371/journal.pntd.0004342)
Supplement: S2 Appendix — (PDF) [file pntd.0004342.s002.pdf]

Enquête sur le DDMS

1. **Des postes suivantes quelle est celle qui correspond mieux à votre emploi principal?**
   Which of the following best describes your job function?
   Mark only one oval.

   - Entomologiste
   - Informaticien
   - Directeur
   - Agent de saisie
   - Gestion des données
   - S&E
   - Other: ....

2. **Dans quel pays est-ce que vous travaillez?**
   What country do you work in?
   Mark only one oval.

   - Benin
   - Éthiopie
   - Guinée Équatoriale
   - Ghana
   - Inde
   - Mali
   - Zambie
   - Other: ....

3. **Avec quelle fréquence est-ce que vous utilisez le DDMS?**
   How often do you use the DDMS?
   Mark only one oval.

   - Jamais
   - Rarement
   - Régulièrement

4. **Utilisant le DDMS me permet d'accomplir plus rapidement des tâches**
   1: Fortement en désaccord; 2: Plutôt pas d'accord; 3: Ni en accord ni en désaccord; 4: Plutôt d'accord; 5: Fortement en accord
   Mark only one oval.

	1	2	3	4	5	
Fortement en désaccord						Fortement en accord

5. **Quelles tâches est-ce que le DDMS vous permetez d'accomplir plus rapidement?**
Cliquez sur "Pas applicable" si vous n'avez pas besoin de compléter ces tâches dans votre position actuelle
Mark only one oval per row.

	Oui	Non	Pas applicable
La saisie des données			
Vérification l'exactitude des données			
Modification/nettoyage des données			
Interrogation / Résumé / Compilation			
Création de graphiques et tableaux			
Création des rapports			
La création de cartes géographiques			
La prise des décisions programmatiques			
Autre			

6. **Si "Autre", décrivez**

7. **Je trouve le DDMS utile pour mon travail**
1: Fortement en désaccord; 2: Plutôt pas d'accord; 3: Ni en accord ni en désaccord; 4: Plutôt d'accord; 5: Fortement en accord
Mark only one oval.

	1	2	3	4	5	
Fortement en désaccord						Fortement en accord

8. **Vous trouvez le DDMS utile pour compléter quelles tâches?**
Cliquez sur "Pas applicable" si vous n'avez pas besoin de compléter ces tâches dans votre position actuelle
Mark only one oval per row.

	Oui	Non	Pas Applicable
La saisie des données			
Vérification l'exactitude des données			
Modification/nettoyage des données			
Interrogation / Résumé / Compilation			
Création de graphiques et tableaux			
Création des rapports			
La création de cartes géographiques			
La prise des décisions programmatiques			
Autre			

9. **Si "Autre", décrivez**

....

10. **Je trouve que le DDMS est facile à utiliser**

1: Fortement en désaccord; 2: Plutôt pas d'accord; 3: Ni en accord ni en désaccord; 4: Plutôt d'accord; 5: Fortement en accord
Mark only one oval.

	1	2	3	4	5	
Fortement en désaccord	◯	◯	◯	◯	◯	Fortement en accord

11. **Quelles composants du DDMS sont faciles à utiliser?**

Cliquez sur "Pas Applicable" si vous n'avez pas utilisé une composant en particulière
Mark only one oval per row.

	Oui	Non	Pas Applicable
Interface générale utilisateur	◯	◯	◯
Écran de saisie de données	◯	◯	◯
Constructeurs de requête / Interrogation des données	◯	◯	◯
Module de cartographie	◯	◯	◯
Outils de reportage	◯	◯	◯
Importation des données	◯	◯	◯
Arbre de géographie	◯	◯	◯
Arbre de termes	◯	◯	◯
Fonctionnalité de sécurité	◯	◯	◯
Constructeur de formulaires	◯	◯	◯
Localisation (changement de langue)	◯	◯	◯

12. **What components of the DDMS do you think could be improved to make them more useful?**

Please place a check mark next to the TOP THREE components
Check all that apply.

- [ ] Écran de saisie de données
- [ ] Constructeurs de requête / Interrogation des données
- [ ] Module de cartographie
- [ ] Outils de reportage
- [ ] Importation des données
- [ ] Arbre de géographie
- [ ] Arbre de termes
- [ ] Fonctionnalité de sécurité
- [ ] Constructeur de formulaires
- [ ] Localisation (changement de langue)
- [ ] Other: ....

13. **Quelles composants du DDMS est-ce que vous trouvez le plus utiles?**
Veuillez crocher LES TROIS composantes le plus utiles selon vous
Check all that apply.

- [ ] Écran de saisie de données
- [ ] Constructeurs de requête / Interrogation des données
- [ ] Module de cartographie
- [ ] Outils de reportage
- [ ] Importation des données
- [ ] Arbre de géographie
- [ ] Arbre de termes
- [ ] Fonctionnalité de sécurité
- [ ] Constructeur de formulaires
- [ ] Localisation (changement de langue)
- [ ] Other: ....

14. **SVP, decrivez comment les composants indiquées ici-dessus pourraient être améliorées**

....
....
....
....
....

15. **Qu'est-ce que vous utilisiez pour organiser vos données brutes avant l'utilisation du DDMS?**
How were you organizing your raw data prior to using the DDMS?
Check all that apply.

- [ ] Feuilles d'Excel
- [ ] Base de données en Access
- [ ] Une autre base de données
- [ ] Papier seulement
- [ ] Other: ....

16. **Comment est-ce que le DDMS compare a cette methode (ci-dessus) en termes de...?**
How does the DDMS compare to this other method in terms of...?
Mark only one oval per row.

	Mieux	Le même	Pire	Je ne sais pas
Qualité des données				
Accès aux données				
Manipulation des données				
Capacité de facilement résumer les données				
La rapidité avec laquelle les tâches connexes aux données sont accomplies				

17. **Seriez-vous intéressé d'utiliser le DDMS pour la gestion d'autres données?**
Mark only one oval.

- Oui
- Non

18. **Pourquoi / Pourquoi pas?**

19. **D'autres commentaires?**
Any additional comments?

Powered by
Google Forms
